# Supplementary material for: Divergent Impact of Enzyme Replacement Therapy on Human Cardiomyocytes and Enterocytes Affected by Fabry Disease: Correlation with Mannose-6-phosphate Receptor Expression
Source: J Clin Med. 2022 Feb 28;11(5):1344. doi: 10.3390/jcm11051344 (PMC8911518; doi:10.3390/jcm11051344)
Supplement: Supplementary file 1 [file jcm-11-01344-s001.zip › jcm-1593944-supplementary.pdf]

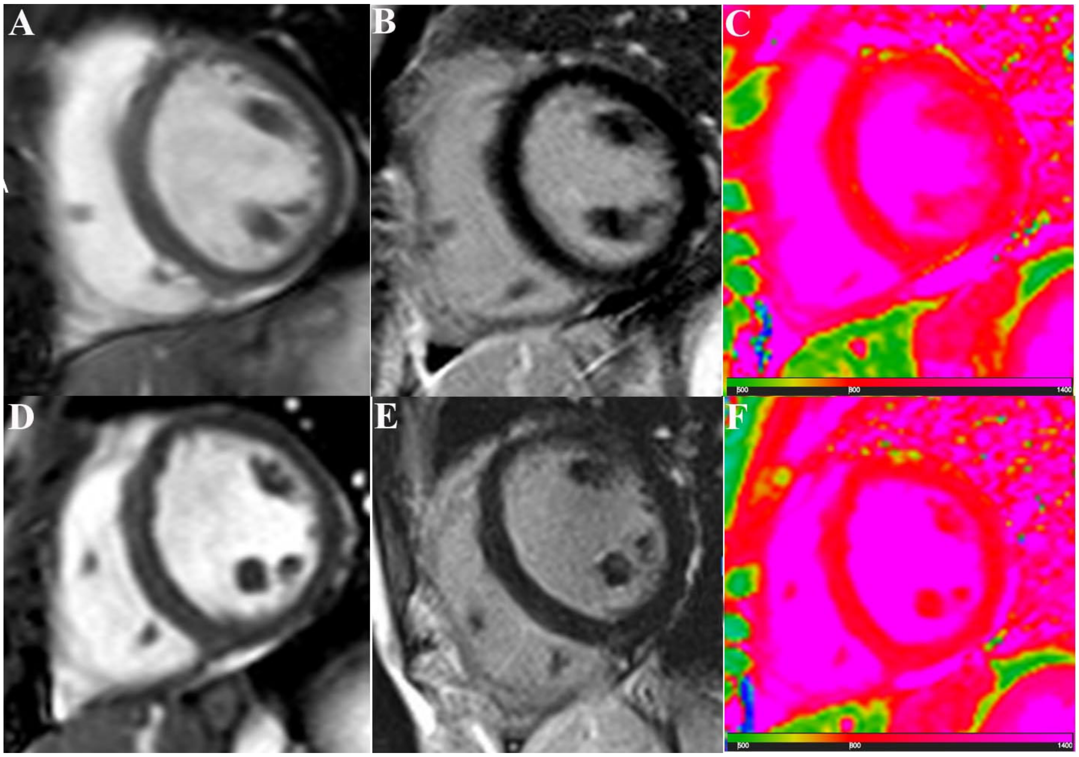

Figure S1. Cardiac Magnetic Resonance in a pre-hypertrophic form of Fabry cardiomyopathy, performed before and after 24-month enzyme replacement therapy in a 29 year-old male.

Before enzymatic therapy, the patient presented with a maximal wall thickness of 9 mm, corresponding to a left ventricular mass of 126 g and normal cardiac contractility (A,B).

T1 mapping performed with MOLLI sequences, showed an average T1 value of 887 msec (C), and was not associated with the presence of late enhancement foci (B).

At follow-up (panel D–F), LV mass and function and native T1 value remained substantially unchanged.

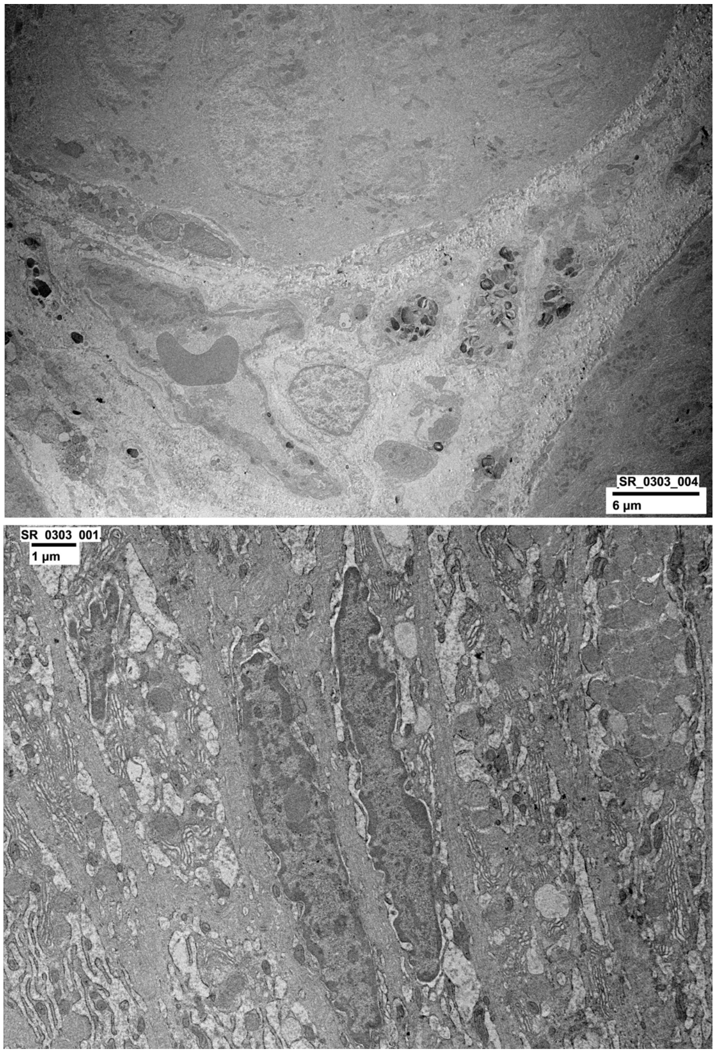

Figure S2 – Subepithelial region of gastric mucosa before and after ERT.

A number of glycolipid bodiea are stored in various cell types, including fibroblsts, endothelial cells and smooth muscele cells of circular muscle layer.

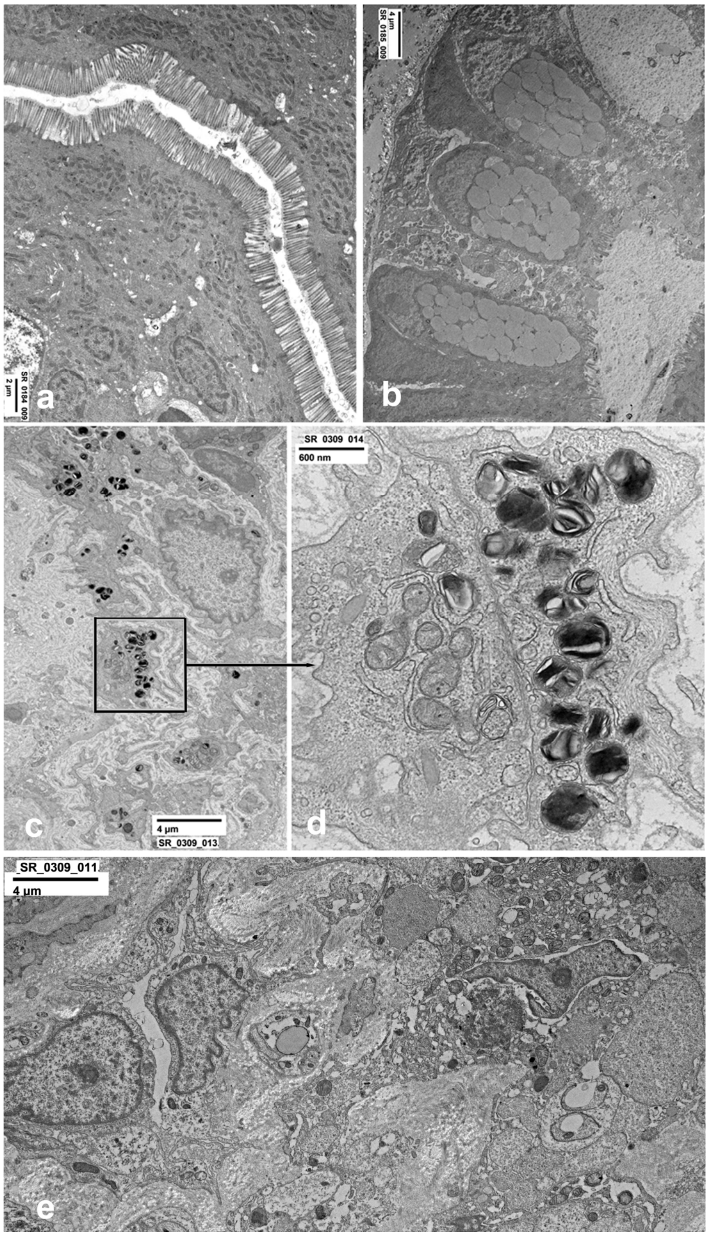

Figure S3- Intestinal (duodenum) cells before and after ERT.

Micrographs show absorptive epithelium (a) and secretive muciparous cells (b) BEFORE ERT. The absence of glycolipid bodies and architecture is similar to that seen AFTER ERT.

(c,d) - Subepithelial region of the duodenal villus before ERT. C – Low magnification micrograph showing the region of the loose connective tissue of the core of the villus BEFORE ERT. Different cell types are visible, containing diffuse glycolipid bodies, in particular, endothelial cells, smooth muscle cell and fibroblasts.

(d) – Detail showing glycolipid bodies stored in the cytoplasm of two cells, possibly fibroblasts.

(e) Subepithelial region similar to figure (c), but AFTER ERT.

To be noted the disappearance of glycolipid bodies in all types of cells.

Table S1 – **Summary of glycolipid vacuole changes in gastrointestinal cells before and after ERT**

Cells	***Stomach***		***Intestine (Duodenum)***	
Treatment	Before ERT	After ERT	Before ERT	After ERT
Epithelial cells	Unaffected		Unaffected	
Goblet muciparous cells	present	Absent or decreased	present	Absent or decreased
Endothelial and smooth muscle cells	present	Occasionally present	present	Occasionally present
Immune cells + macrophages	Occasionally present	Absent or decreased		absent
Fibroblasts	present	absent	present	absent
Endocrine	Unaffected		Unaffected	
Gangliar cells	Unaffected		Unaffected	
